# Extracellular Matrix Stiffness Regulates Osteogenic Differentiation through MAPK Activation

**DOI:** 10.1371/journal.pone.0135519

**Published:** 2015-08-11

**Authors:** Jun-Ha Hwang, Mi Ran Byun, A. Rum Kim, Kyung Min Kim, Hang Jun Cho, Yo Han Lee, Juwon Kim, Mi Gyeong Jeong, Eun Sook Hwang, Jeong-Ho Hong

**Affiliations:** 1 Department of Life Sciences, School of Life Sciences and Biotechnology, Korea University, Seoul, Korea; 2 College of Pharmacy and Graduate School of Pharmaceutical Sciences, Ewha Womans University, Seoul, Korea; University of California, San Diego, UNITED STATES

## Abstract

Mesenchymal stem cell (MSC) differentiation is regulated by the extracellular matrix (ECM) through activation of intracellular signaling mediators. The stiffness of the ECM was shown to be an important regulatory factor for MSC differentiation, and transcriptional coactivator with PDZ-binding motif (TAZ) was identified as an effector protein for MSC differentiation. However, the detailed underlying mechanism regarding the role of ECM stiffness and TAZ in MSC differentiation is not yet fully understood. In this report, we showed that ECM stiffness regulates MSC fate through ERK or JNK activation. Specifically, a stiff hydrogel matrix stimulates osteogenic differentiation concomitant with increased nuclear localization of TAZ, but inhibits adipogenic differentiation. ERK and JNK activity was significantly increased in cells cultured on a stiff hydrogel. TAZ activation was induced by ERK or JNK activation on a stiff hydrogel because exposure to an ERK or JNK inhibitor significantly decreased the nuclear localization of TAZ, indicating that ECM stiffness-induced ERK or JNK activation is important for TAZ-driven osteogenic differentiation. Taken together, these results suggest that ECM stiffness regulates MSC differentiation through ERK or JNK activation.

## Introduction

The extracellular matrix (ECM) is a dynamic structure that provides structural support for organs and tissues. It closely contacts cells, activates several cellular components, and regulates cell proliferation, differentiation, and migration [1]. The chemical composition and structure of the ECM, which are unique to each tissue, are important for cell-ECM interaction and cellular function. Dysregulation of ECM composition, structure, and stiffness contribute to diverse pathological conditions [2]. In particular, ECM stiffness regulates multipotent mesenchymal stem cell (MSC) differentiation; a soft matrix has neurogenic potential, and a stiff matrix that mimics collagenous bone has osteogenic potential [3–6]. It has also been shown that stiff matrix-driven osteogenesis was induced by integrin-mediated mechanotransduction [7].

Transcriptional coactivator with PDZ-binding motif (TAZ) and its paralog Yes associated protein (YAP) were characterized as signaling mediators of mechanotransduction [8–10]. The activity of TAZ and YAP are regulated by ECM stiffness [8]. A stiff ECM stimulates nuclear localization of TAZ/YAP and facilitates osteogenic differentiation, whereas a soft ECM inhibits their nuclear localization and induces adipogenic differentiation [8,11]. A stiff ECM activates Rho GTPase, which stimulates F-actin polymerization and activates TAZ and YAP [8]. Mechanical forces are an important regulators of TAZ/YAP activity [10,12].

TAZ regulates MSC differentiation by activating osteoblast and myoblast differentiation and inhibiting adipocyte differentiation [13–15]. TAZ stimulates Runx2 target genes, but inhibits PPARγ-mediated gene transcription [13]. TAZ and YAP are also known as effector proteins in the Hippo signaling pathway, which plays an important role in cell proliferation, tumorigenesis, and stem cell self-renewal [16,17]. In the present study, we show that a stiff ECM induces ERK and JNK activation, facilitates the nuclear localization of TAZ, and stimulates osteogenic differentiation.

## Results

### A stiff surface stimulates the nuclear localization of TAZ, a mechanotransduction effector

**Fig 1:**
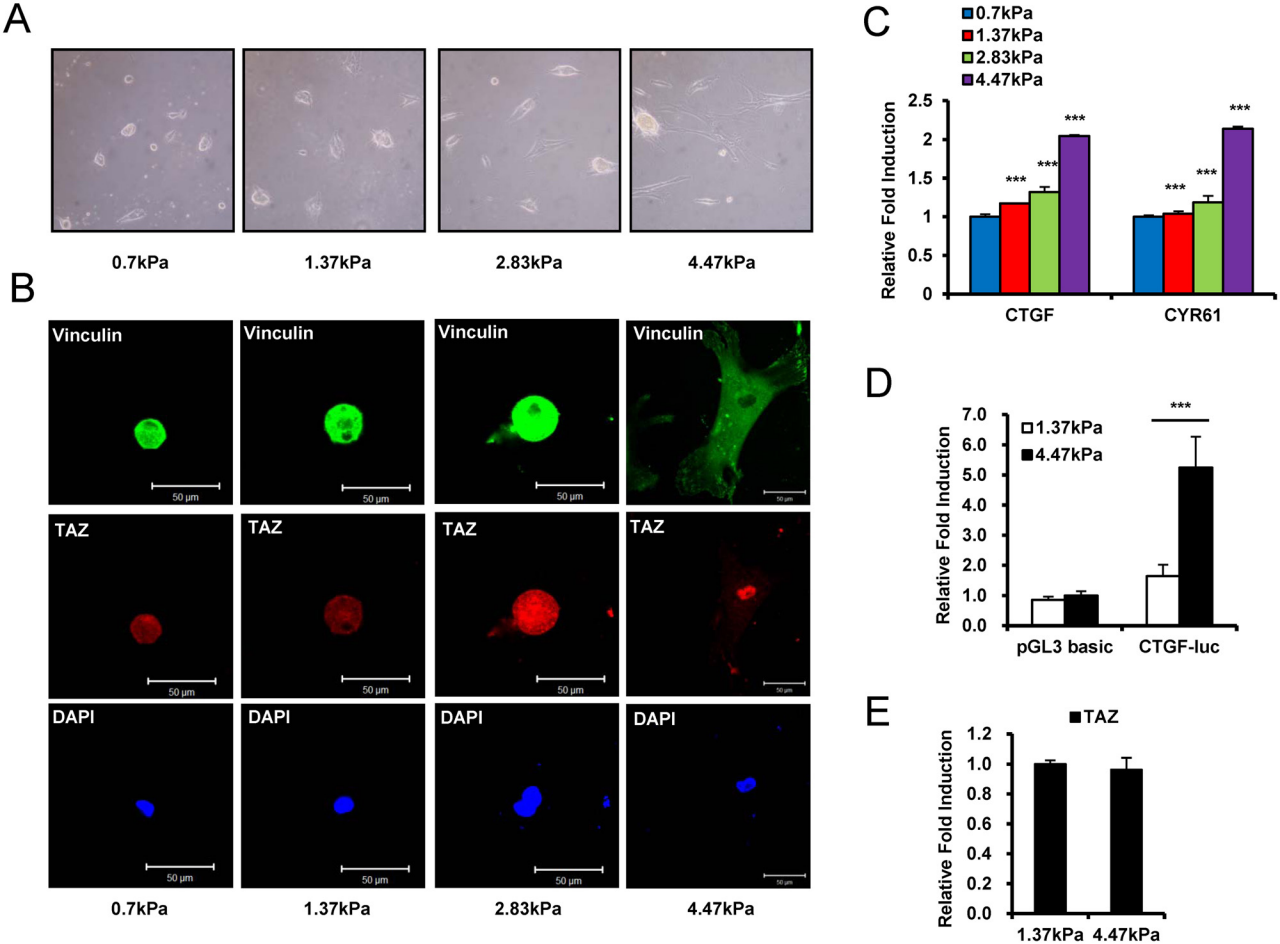
ECM stiffness regulates the cellular phenotype of human mesenchymal stem cells (hMSCs) and the localization and transcriptional activity of TAZ. (A) In indicated hydrogels, cell adhesion and morphology were visualized by light microscopy. Bright field images were taken 24hr after seeding. (B) ECM stiffness controls focal adhesion complex formation and TAZ localization. hMSCs were immunostained with an anti-vinculin antibody to detect focal adhesions (green fluorescence signal) 24 h after seeding. TAZ localization was visualized as a red fluorescence signal. DAPI was used to stain the cell nucleus. (C) The expression of TAZ target genes, including *CTGF* and *CYR61*, were analyzed by qRT-PCR using the cells in panel (A). Target gene expression was normalized to the *GAPDH* expression. Data is shown as fold induction. Asterisks indicate statistical significance (***p < 0.005, t-test). (D) The luciferase reporter gene CTGF-luc was introduced into hMSCs. After 16 h, the transfected cells were plated on 1.37 or 4.47 kPa hydrogels. After 24 h, luciferase reporter gene activity was analyzed. The pGL3-basic luciferase reporter gene, which has no promoter for transcription, was used as a negative control. A Renilla luciferase-expressing vector was used as a transfection control. Luciferase activity was normalized to Renilla luciferase activity and is expressed as relative fold induction. (***p < 0.005, t-test). (E) hMSCs were seeded on a 1.37 or 4.47 kPa hydrogel, and twenty four hours after seeding, total RNAs were isolated and qRT-PCR analysis was assessed to see the expression of *TAZ* gene. Gene expression was normalized to *GAPDH*.

Tissues have diverse elasticity values; normal liver and brain have values of several hundred Pascals (Pa), whereas muscle has a value of more than 12 kPa, and tendon and cartilage have values in the megapascal range [7]. To study the function of TAZ on a stiff ECM and identify the minimum stiffness required for TAZ activation in tissues, TAZ localization was analyzed in tissues grown on hydrogels with various degrees of stiffness by immunocytochemistry. Previously, increased TAZ nuclear localization was observed in cells on a 40 kPa gel matrix, but not on a 0.7 kPa matrix [8]. However, the difference was too extreme to define the minimal stiffness required for TAZ activation. Thus, we attempted to determine the minimal stiffness of gel matrix required for the nuclear localization of TAZ. We analyzed the nuclear localization of TAZ in cells on 0.7, 4.47, 8.73, and 40 kPa hydrogels by immunocytochemistry, which was evidenced by the detection of a green fluorescence signal for TAZ in the nucleus. We observed that TAZ was localized to the nucleus on hydrogels with stiffnesses greater than 4.47 kPa (S1 Fig). Next, we narrowed down the stiffness range by assessing nuclear localization on 4.47, 2.83, 1.37, and 0.7 kPa hydrogels. Eventually, we observed that a hydrogel matrix with a stiffness of 4.47 kPa is required for TAZ activation, as no activation was observed on hydrogel matrices with stiffnesses less than 2.8 kPa (Fig 1A and 1B). In addition, cells with normal spread shape and focal adhesions were observed on the 4.47 kPa hydrogel matrix, as shown by vinculin staining (Fig 1A and 1B, S1 Fig).

Next, to assess the function of TAZ on these hydrogel matrices, we measured the transcriptional activity of TAZ. The expression of two TAZ target genes, *CTGF* and *CYR61*, was analyzed by qRT-PCR. As shown in Fig 1C, the 4.47 kPa hydrogel significantly stimulated *CTGF* and *CYR61* expression (by 2 fold) compared to the expression levels observed in cells on the 0.7 kPa hydrogel matrix. To further investigate the transcriptional activity of TAZ, the CTGF promoter, which contains TEADs transcription factor binding sites, was fused to a luciferase reporter gene (CTGF-Luc) and was transfected into MSCs, and the cells were plated on 4.47 and 1.37 kPa hydrogels. We observed that the 4.47 kPa hydrogel significantly stimulated the transcription of CTGF-Luc (Fig 1D). TAZ transcription was not different in the two hydrogels, indicating that hydrogel stiffness does not regulate TAZ transcription (Fig 1E). These results suggest that approximately 4 kPa is the minimal stiffness required for the nuclear localization and activation of TAZ.

### A stiff hydrogel matrix stimulates osteogenic differentiation, but inhibits adipogenic differentiation

**Fig 2:**
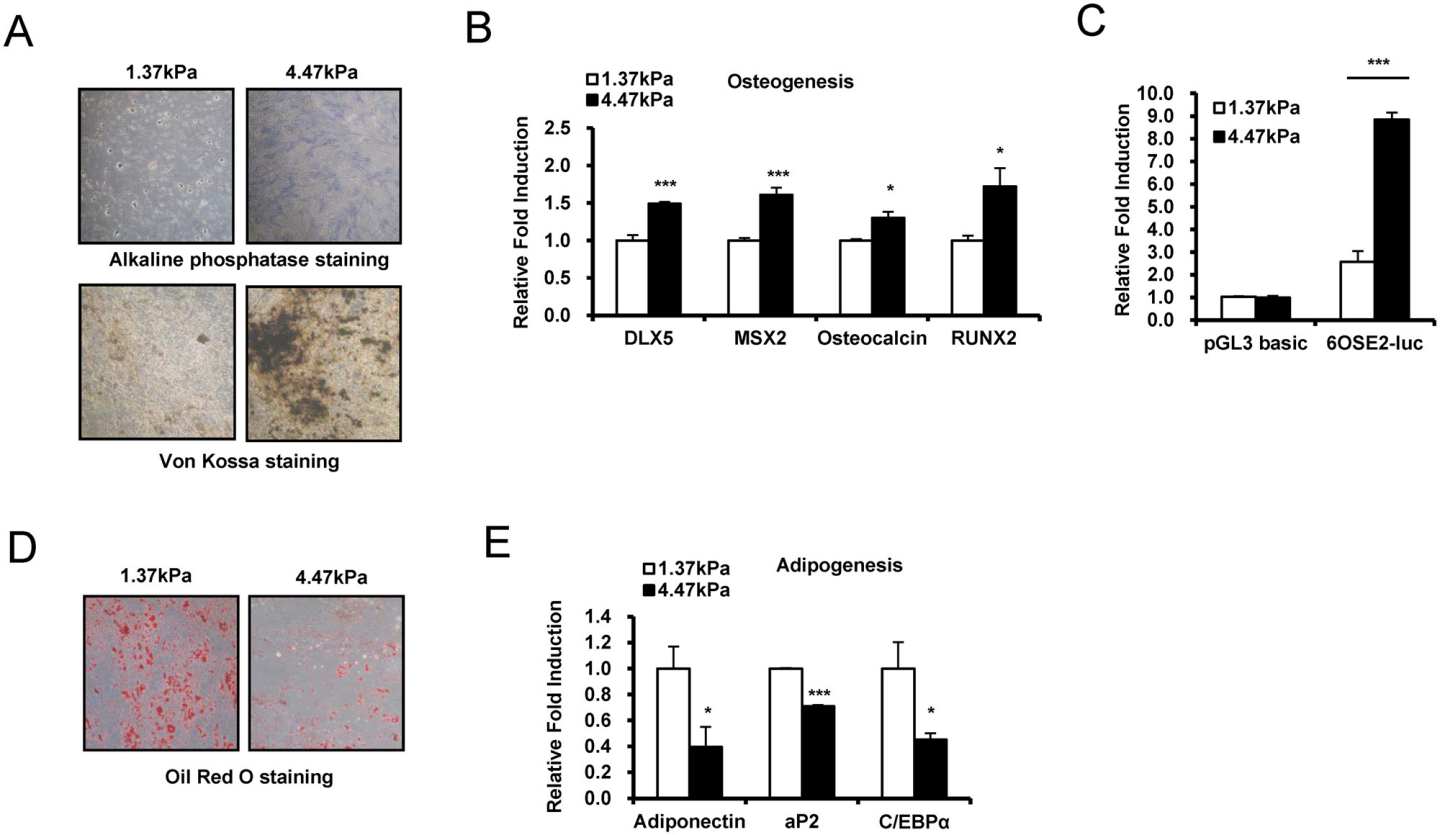
ECM stiffness stimulates osteoblast differentiation and represses adipocyte differentiation. (A) A stiff hydrogel stimulates osteogenic differentiation. hMSCs were seeded on a 1.37 or 4.47 kPa hydrogel, and osteogenic differentiation was induced 24 h after seeding. Cells were stained for alkaline phosphatase at 9 days after differentiation and Von Kossa staining was assessed to see mineralization at 21 days after differentiation. (B) qRT-PCR analysis of the osteoblast marker genes *DLX5*, *MSX2*, osteocalcin, and *RUNX2* of the cells used in panel (A). Expression of the marker genes was normalized to *GAPDH* expression. Data are presented as fold induction. (*p < 0.05, ***p < 0.005, t-test) (C) The luciferase reporter gene construct 6OSE2-luc was introduced into hMSCs along with a Runx2-expressing vector, which provides similar amounts of Runx2 protein in assay condition. After 16 h, the transfected cells were plated on a 1.37 or 4.47 kPa hydrogel. After 24 h, luciferase reporter gene activity was analyzed. The pGL3-basic luciferase reporter gene construct, which has no promoter for transcription, was used as a negative control. A Renilla luciferase-expressing vector was used as a transfection control. Luciferase activity was normalized to Renilla luciferase activity and is expressed as relative fold induction. (***p < 0.005, t-test) (D) hMSCs were seeded on a 1.37 or 4.47 kPa hydrogel, and adipogenic differentiation was induced 24 h after seeding. Oil Red O staining was assessed to see lipid droplet at 9 days after differentiation (E) The expression of the adipogenic marker genes adiponectin, *aP2*, and *C/EBPα* were analyzed by qRT-PCR in adipocyte-differentiated hMSCs. Adipogenic differentiation was assessed according to the protocol described in the Materials and Methods. Target gene expression was normalized to *GAPDH*. Data are expressed as relative fold induction. (*p < 0.05, ***p < 0.005, t-test)

TAZ interacts with Runx2 and stimulates osteoblast differentiation of MSCs [13]. Because we observed preferential nuclear localization and transcriptional activation of TAZ on the 4.47 kPa hydrogel matrix, we next determined whether the 4.47 kPa hydrogel matrix can stimulate the osteogenic differentiation of MSCs. MSCs cultured on a 4.47 or 1.37 kPa hydrogel matrix were incubated with osteogenic differentiation medium, and differentiation potential was analyzed as alkaline phosphatase activity and Von Kossa staing, a marker of osteogenic differentiation. As shown in Fig 2A, cells cultured on a 4.47 kPa hydrogel matrix showed significantly higher alkaline phosphatase activity and increased mineralization by Von Kossa staining than cells cultured on a 1.37 kPa hydrogel. To further assess osteogenic potential, total RNA was isolated, and the expression of the osteogenic differentiation marker genes *DLX5*, *MSX2*, osteocalcin, and *RUNX2* were analyzed by qRT-PCR. The data in Fig 2B show that the marker genes were significantly induced in cells cultured on the 4.47 kPa hydrogel matrix. To further assess transcriptional activity on a 4.47 kPa hydrogel, MSCs were transfected with a luciferase reporter gene construct containing a Runx2 binding site (6OSE2-luc), and reporter gene activity was measured in cells cultured on a 1.37 or 4.47 kPa hydrogel. As shown in Fig 2C, higher reporter gene activity was observed in cells on 4.47 kPa hydrogels than in cells on 1.37 kPa hydrogels, indicating that stiffer hydrogels stimulate Runx2-mediated gene transcription. These results suggest that the 4.47 kPa hydrogel matrix stimulates osteogenic differentiation of MSCs when compared to that observed on the 1.37 kPa hydrogel.

Next, we determined whether stiff hydrogels can regulate the fate of human MSCs (hMSCs) by inhibiting adipogenic differentiation while stimulating osteogenic differentiation. hMSCs were incubated with adipogenic differentiation medium on a 4.47 or 1.37 kPa hydrogel matrix. As shown in Fig 2D and 2E, lower adipogenic potential was observed in cells on the 4.47 kPa hydrogel than in cells on the 1.37 kPa hydrogel, as indicated by the decreased fat droplet by Oil Red O staining (Fig 2D) and decreased expression of adipogenic marker genes, including adiponectin, *aP2*, and *C/EBPα* (Fig 2E). These results suggest that ECM stiffness plays an important role in the fate decision of MSCs.

### ERK and JNK kinases are important for stiff hydrogel-mediated osteogenic differentiation

**Fig 3:**
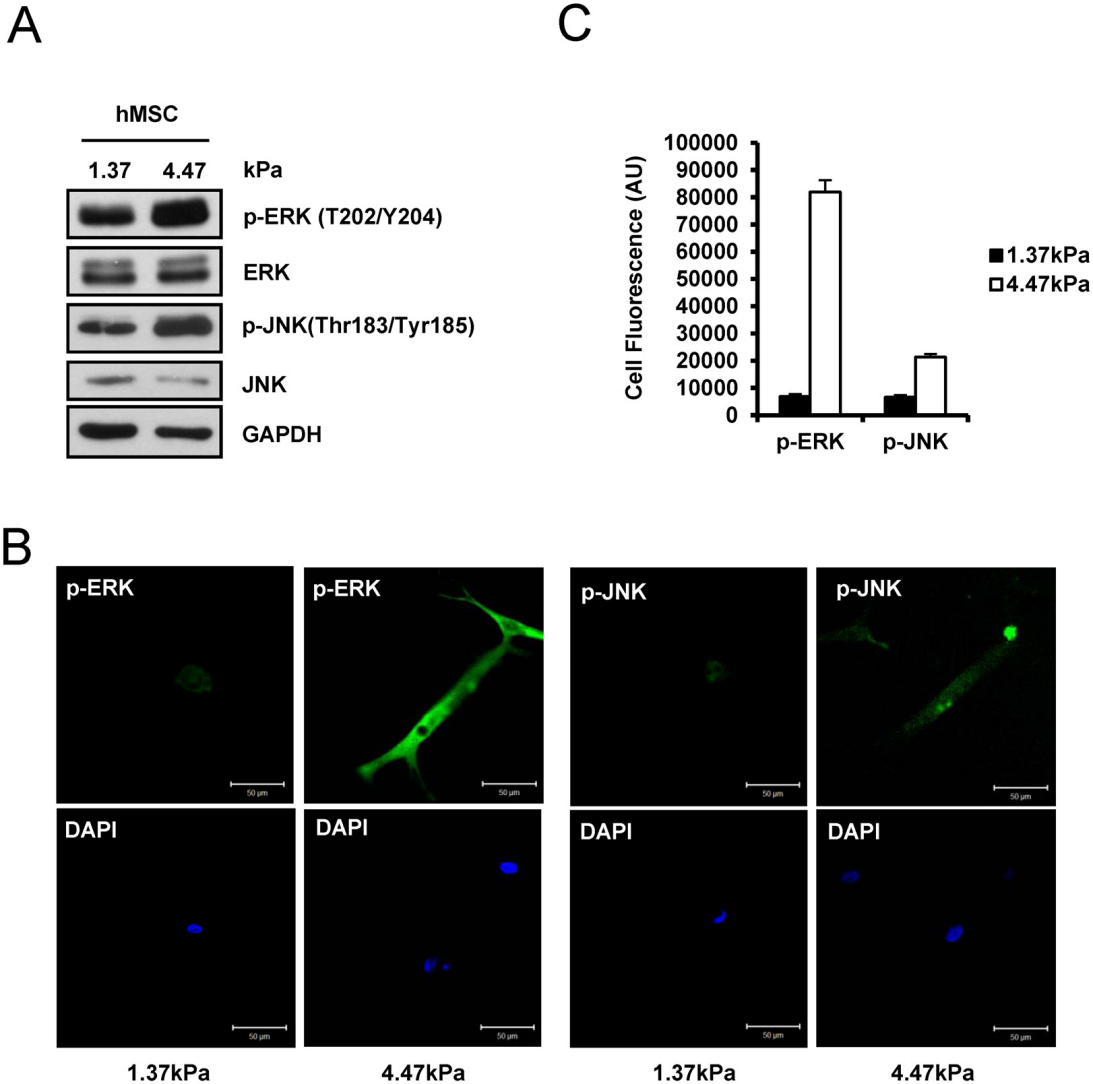
A stiff ECM activates the ERK and JNK signaling pathway. (A) hMSCs were plated on a 1.37 or 4.47 kPa hydrogel, and after 12 h, cell lysates were prepared and analyzed by immunoblotting. To assess the activity of ERK and JNK, phosphorylated ERK (p-ERK) and phosphorylated JNK (p-JNK) antibodies were used, respectively. As a control, total ERK and JNK protein was analyzed. The levels of phosphorylated ERK and JNK were increased in hMSCs cultured on the stiff matrix. GAPDH was used as a loading control. (B) Immunocytochemistry of p-ERK and p-JNK in hMSCs on a 1.37 or 4.47 kPa hydrogel. Cells in panel (A) were fixed and subjected to immunocytochemical staining with a p-ERK or p-JNK antibody. The signals for p-ERK or p-JNK were green fluorescence. DAPI was used for nuclear staining. (C) Fluorescence signals in panel (B) was quantified by image J software and corrected total cell fluorescence was calculated by fluorescence signal with elimination of background signal. AU is arbitrary unit.

MAPKs are important signaling kinases for osteogenic differentiation [18]. We also observed that TAZ activity and nuclear localization are significantly increased by ERK activation [19,20]. Thus, we studied whether MAPK activity is important for stiffness mediated cellular function. For the experiments, we analyzed ERK and JNK activity by immunoblot analysis. As shown in Fig 3A, the levels of phosphorylated ERK and JNK were significantly higher in cells cultured on the 4.47 kPa hydrogel matrix than in cells cultured on the 1.37 kPa hydrogel matrix even though the levels of total ERK and JNK protein are similar in cells cultured on the two hydrogels. Similar results were observed in the immunocytochemical study and its quantification (Fig 3B and 3C). The results show that ERK and JNK are activated on the 4.47 kPa hydrogel matrix and suggest that ERK and JNK are important signaling mediators for stiff hydrogel-mediated mechanotransduction.

**Fig 4:**
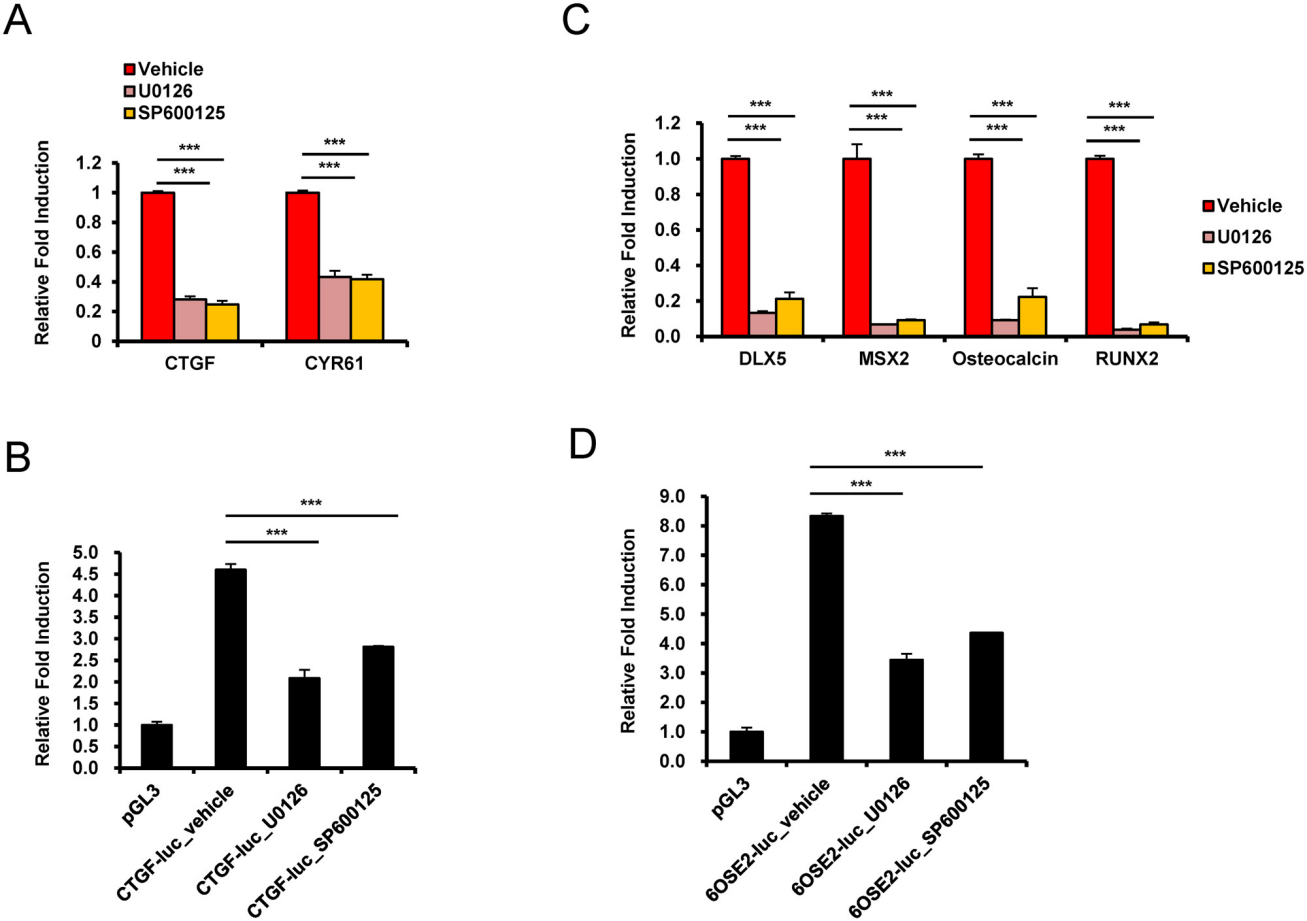
Stiffness-regulated ERK/JNK activity is crucial for TAZ target gene expression and osteogenesis at the transcriptional level. (A) hMSCs cultured on the 4.47 kPa hydrogel were treated with a MEK inhibitor (U0126, 10 μM) or a JNK inhibitor (SP600125, 10 μM). After 12 h, total RNA was prepared, and *CTGF* and *CYR61* expression was assessed by qRT-PCR. The results showed that *CTGF* and *CYR61* were downregulated following inhibition of ERK or JNK in cells on the stiff hydrogel. (B) The CTGF-luc reporter gene construct or the control pGL3-basic vector was transfected into hMSCs, and after 16 h, the transfected cells were plated on 4.47 kPa hydrogels. After 24 h, luciferase reporter gene activity was analyzed. To inhibit ERK or JNK, cell were pretreated with 10 μM U0126 or 10 μM SP600125, respectively, 12 h before reporter gene analysis. A Renilla luciferase-expressing vector was used as a transfection control. Luciferase activity was normalized to Renilla luciferase activity. (C) hMSCs were differentiated into osteoblasts for 6 days in the presence of 10 μM U0126 or 10 μM SP600125. DMSO was used as the vehicle control. The expression of osteoblastic marker genes, including *DLX5*, *MSX2*, osteocalcin, and *RUNX2*, were analyzed by qRT-PCR. Gene expression was normalized to *GAPDH*. The results show that the expression of the osteogenic marker genes in cells on a 4.47 kPa hydrogel was significantly suppressed by ERK or JNK inhibition. (D) hMSCs were transfected with 6OSE2-luc or pGL3-basic (control) along with a Renilla luciferase-expressing construct. Then, the cells were plated on a 4.47 kPa hydrogel, and after 24 h, luciferase reporter gene activity was analyzed. To inhibit ERK or JNK, cells were pretreated with 10 μM U0126 or 10 μM SP600125, respectively, 12 h before the reporter gene assay. Reporter gene luciferase activity was normalized to Renilla luciferase activity. (***p < 0.005, t-test)

Next, to further examine the importance of ERK and JNK activity, cells cultured on the 4.47 kPa hydrogel were treated with the ERK signaling inhibitor U0126 (specifically, a MEK inhibitor) or the JNK inhibitor SP60015, and the expression of the TAZ target genes *CTGF* and *CYR61* was examined. As shown in Fig 4A, expression of both target genes was significantly inhibited by the ERK and JNK inhibitors. To determine whether CTGF suppression induced by the ERK or JNK inhibitor is regulated by TEADs transcriptional activation, the CTGF-luc construct was introduced into MSCs, and then the cells were seeded on a 4.47 kPa hydrogel and treated with the ERK or JNK inhibitor. As shown in Fig 4B, the ERK and JNK inhibitors suppressed CTGF-luc reporter gene expression, suggesting that TEADs-mediated gene transcription is regulated by ERK and JNK.

Next, we investigated the osteogenic effects of ERK and JNK in a stiff hydrogel matrix. Osteogenic differentiation of cells on the 4.47 kPa hydrogel was induced in the presence of the ERK or JNK inhibitor. As shown in Fig 4C, the stiff hydrogel-induced osteogenic marker gene expression was significantly suppressed in the presence of the ERK or JNK inhibitor (Fig 4C). To understand the mechanism underlying osteogenic marker gene expression, 6OSE2-luc was introduced into MSCs, and the cells were cultured on the 4.47 kPa hydrogel and treated with the ERK or JNK inhibitor. As shown in Fig 4D, the ERK and JNK inhibitor suppressed 6OSE2-Luc reporter gene expression, suggesting that Runx2-mediated gene transcription is regulated by ERK and JNK. Taken together, these results suggest that ERK and JNK play an important role in stiff hydrogel-induced TAZ target gene activation.

### ERK and JNK are important for the nuclear localization of TAZ

**Fig 5:**
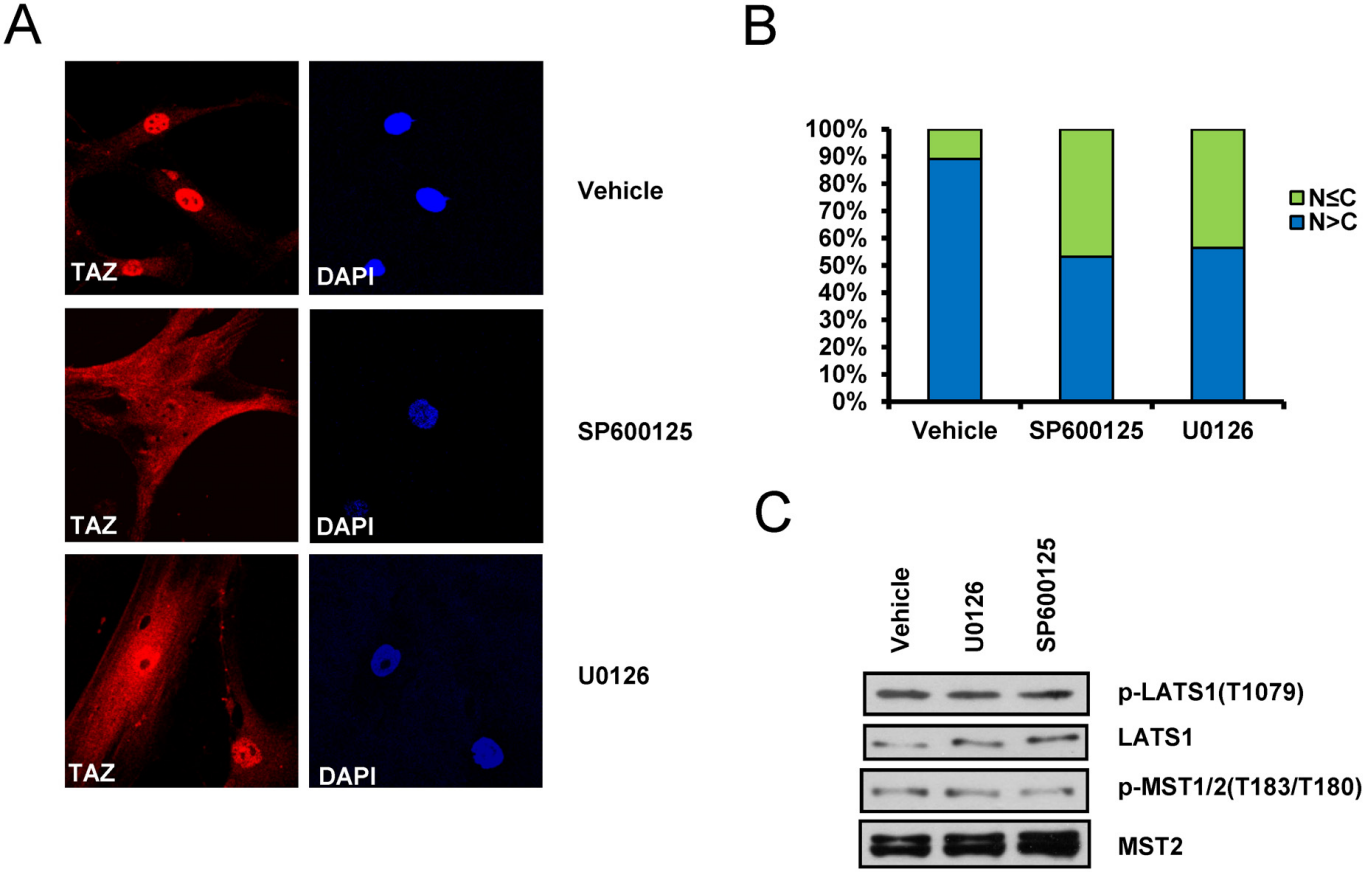
Inhibition of the ERK or JNK signaling pathway induces TAZ cytoplasmic localization on stiff hydrogels. (A) hMSCs on a 4.47 kPa hydrogel were treated with 10 μM U0126 or 10 μM SP600125. After 12 h of treatment, cells were subjected to immunostaining with an anti-TAZ antibody. The red fluorescence signal shows the location of TAZ, and DAPI was used to stain the nuclei. The results show that even in cells on a stiff matrix, TAZ is localized evenly to the cytoplasm and nucleus following ERK or JNK inhibition. (B) Approximately 100 cells in panel (A) were counted, and TAZ localization was analyzed in these cells. The counting procedure was done using the Image J program. The number of cells that showed an even cytoplasmic-nuclear or cytoplasm-dominant TAZ localization was higher in the presence of an ERK or JNK inhibitor than in the absence of either inhibitor. (C) Cell lysates in panel (A) were prepared, and the activity of the Hippo signaling pathway components LATS and MST kinase was analyzed by immunoblotting. The phosphorylation status of LATS and MST kinase was analyzed with p-LATS1 and p-MST1/2 antibodies, respectively. Total LATS1 and MST2 levels were detected as a loading control.

To study whether stiff hydrogel-induced TAZ activation is regulated by ERK and JNK activation, MSCs cultured on 4.47 kPa hydrogels were treated with the ERK or JNK inhibitor, and then the nuclear localization of TAZ was analyzed by immunocytochemistry. As shown in Fig 5A and 5B, in cells on the 4.47 kPa hydrogel, TAZ nuclear localization was observed; however, when the cells were treated with the ERK or JNK inhibitor, the cell population with nuclear-localized TAZ decreased significantly, by approximately 40%. These results suggest that ERK and JNK play important roles in the nuclear localization and activation of TAZ.

TAZ functions as an effector of the Hippo signaling pathway, which is involved in organ size control, cell proliferation, and differentiation. The Hippo signal inhibits the nuclear localization of TAZ, which interacts with TEAD transcription factors and stimulates *CTGF* and *CYR61* [17]. In our study, inhibition of ERK or JNK did not perturb the Hippo signaling molecules MST and LATS kinase, which induce TAZ phosphorylation and inhibit TAZ nuclear localization, because we did not observe activation of MST1/2 and LATS1 in cells on the stiff ECM hydrogel after addition of a ERK or JNK inhibitor (Fig 5C). Thus, it seems that TAZ activation through ERK and JNK occurs via other mechanisms and not through alteration of Hippo signaling.

### Rho activation induced TAZ target gene expression on a stiff hydrogel matrix

**Fig 6:**
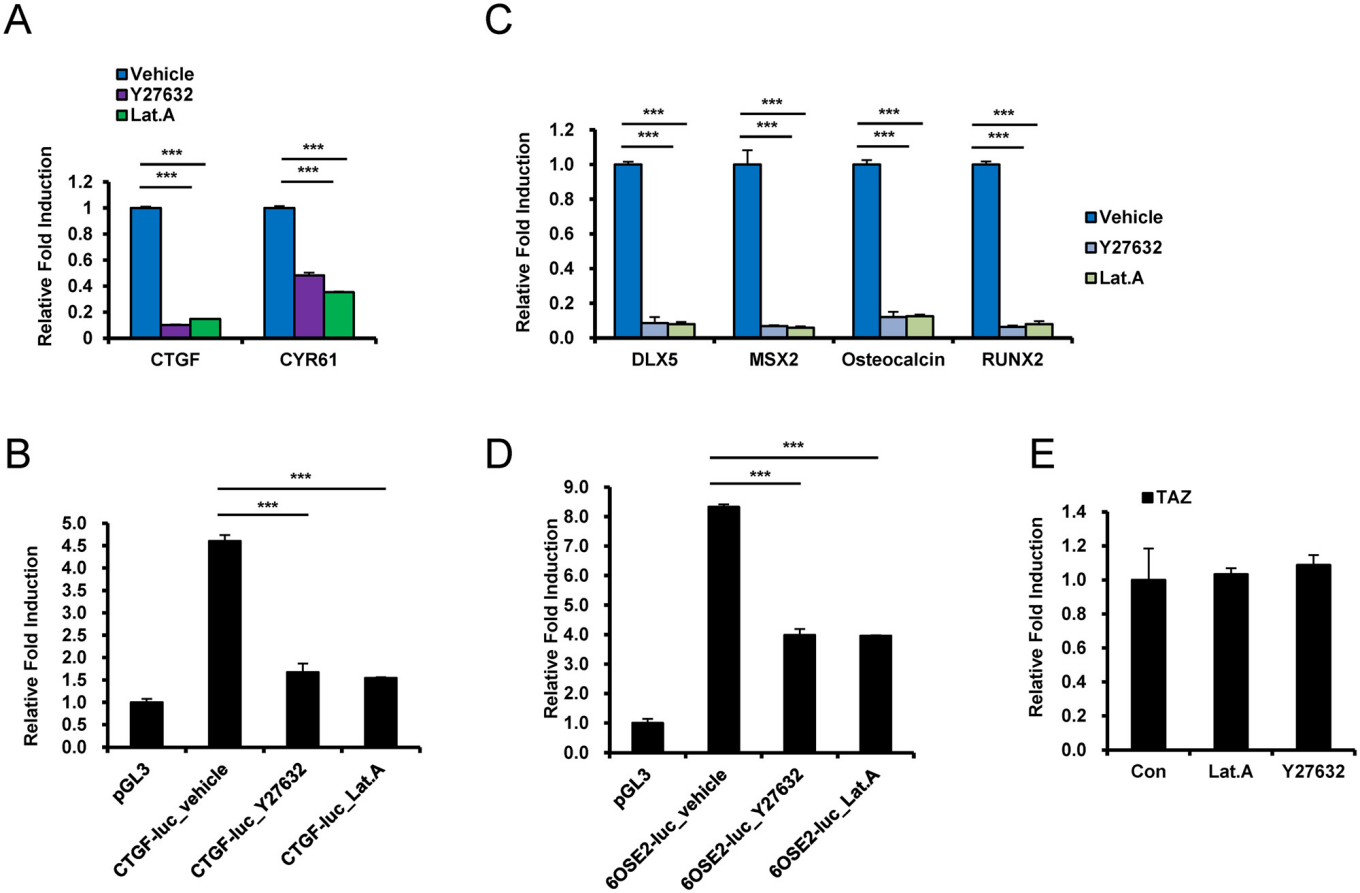
Inhibition of ROCK/F-actin represses the transcriptional activity of TAZ in hMSCs. (A) hMSCs cultured on a 4.47kPa hydrogel were treated with a ROCK inhibitor (Y27632, 50 μM) or an F-actin inhibitor (latrunculin A, 0.5 μM). After 12 h of treatment, total RNA was prepared, and *CTGF* and *CYR61* expression was assessed by qRT-PCR. (B) The CTGF-luc reporter gene construct or the pGL3-basic control vector was transfected into hMSCs, and after 16 h, the transfected cells were plated on 4.47 kPa hydrogels. After 24 h, luciferase reporter gene activity was analyzed. To inhibit ROCK or F-actin, cells were pretreated with 50 μM Y27632 or 0.5 μM latrunculin A 12 h before reporter gene analysis. A Renilla luciferase-expressing vector was used as a transfection control. Luciferase activity was normalized to Renilla luciferase activity. (C) hMSCs on 4.47 kPa hydrogels were differentiated into osteoblasts for 6 days in the presence of 50 μM Y27632 or 0.5 μM latrunculin A. DMSO was used as the vehicle control. The expression of osteoblastic marker genes, including *DLX5*, *MSX2*, osteocalcin, and *RUNX2*, were analyzed by qRT-PCR. Target gene expression was normalized to *GAPDH*. (D) hMSCs were transfected with 6OSE2-luc or pGL3-basic (control) along with a Renilla luciferase-expressing construct. Then, the cells were plated on a 4.47 kPa hydrogel. After 24 h, luciferase reporter gene activity was analyzed. To inhibit ROCK or F-actin, the cells were pretreated with 50 μM Y27632 or 0.5 μM latrunculin A 12 h before the reporter gene assay. Luciferase activity was normalized to Renilla luciferase activity. (***p < 0.005, t-test). (E) Total RNAs of hMSCs in panel (A) were prepared and qRT-PCR was assessed to analyze the transcription of *TAZ*. Gene expression was normalized to *GAPDH*.

It was previously shown that Rho activation is important for mechanotransduction and stimulates actin polymerization and TAZ activation [21,22]. Thus, we determined whether the Rho signal is important for TAZ target gene activation on a 4.47 kPa hydrogel matrix. For these experiments, cells on a 4.47 kPa hydrogel matrix were treated with Y27632 (an inhibitor of Rock, a downstream kinase of Rho) or latrunculin A (an actin polymerization inhibitor). As shown in Fig 6A, latrunculin A and Y27632 blocked TAZ target gene activation in cells on the 4.47 kPa hydrogel matrix. Next, to determine whether CTGF suppression is regulated by TEADs transcriptional activation, CTGF-luc containing MSCs on a 4.47kPa hydrogel were treated with Y27632 or latrunculin A. As shown in Fig 6B, both Y27632 and latrunculin A suppressed CTGF-Luc reporter expression, indicating that TAZ-TEADs-induced transcriptional activation is regulated by Rho activation and actin polymerization.

Next, we also analyzed the effects of Y27632 and latrunculin A during osteogenic differentiation. As shown in Fig 6C, treatment with Y27632 or latrunculin A decreased the osteogenic marker gene expression that was induced by culturing on the 4.47 kPa hydrogel. To further understand the mechanism of osteogenic marker gene expression, 6OSE2-luc-containing MSCs cultured on the 4.47 kPa hydrogel were treated with Y27632 or latrunculin A. As shown in Fig 6D, both inhibitors suppressed 6OSE2-luc reporter expression, suggesting that Runx2-mediated gene transcription is regulated by Rho activation and actin polymerization. TAZ transcription was not changed in the presence of Y27632 or latrunculin A (Fig 6E). These results suggest that Rho activation and actin polymerization are important for stiff hydrogel-induced osteogenic activation.

Taken together, our results show that a stiff (4.47 kPa) hydrogel induces TAZ activation and osteogenic differentiation of MSCs and that ERK and JNK activation is important for stiff hydrogel-mediated TAZ activation.

## Discussion

**Fig 7:**
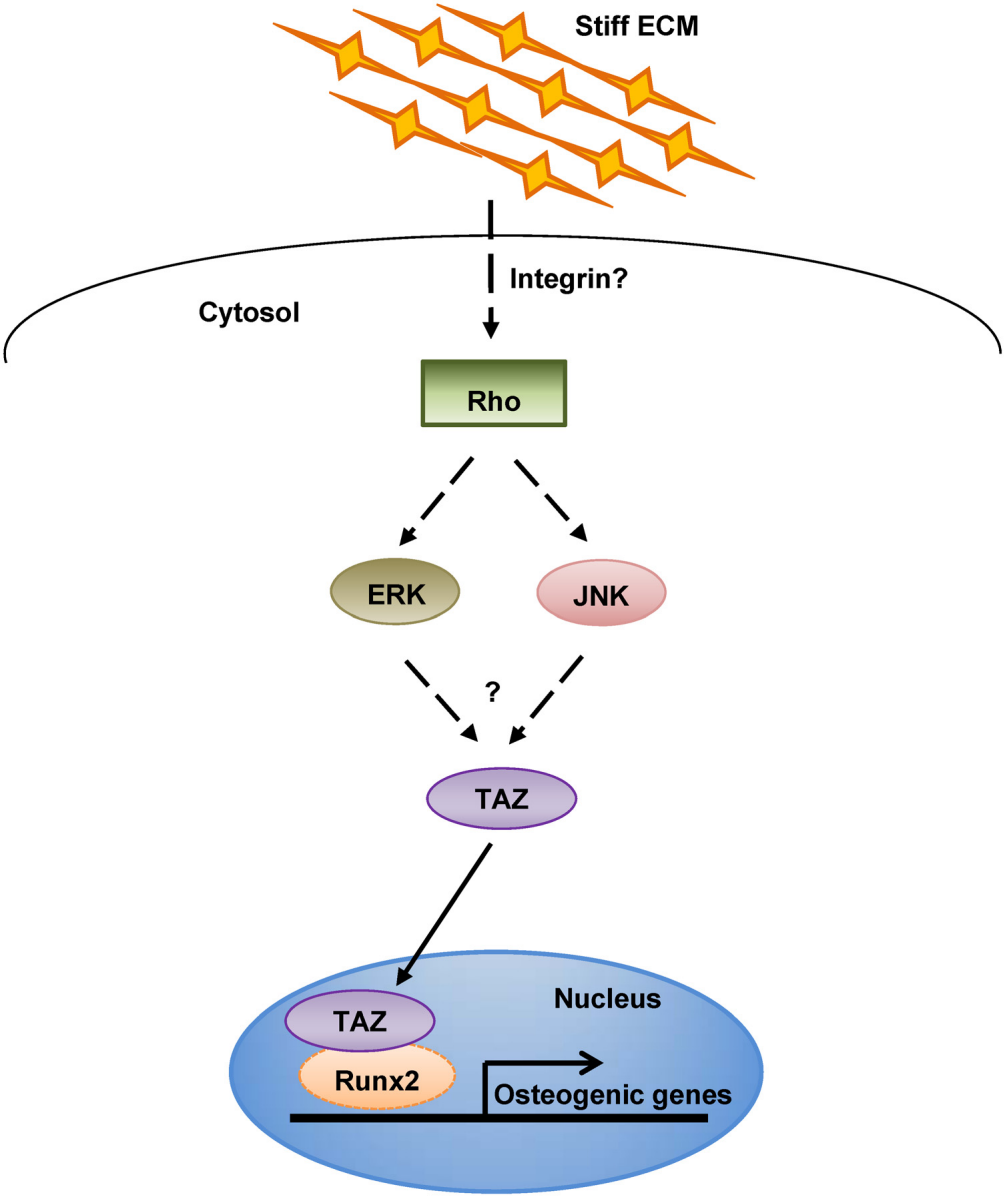
Experimental model. Upon exposure to a stiff ECM environment, the Rho signaling pathway is activated, and ERK or JNK signaling is induced. Activated ERK or JNK promotes the nuclear localization of TAZ and activates the expression of TAZ target genes, including RUNX2-mediated osteoblastic marker genes.

The ECM mediates diverse cellular functions, including cell differentiation. In our study, the biological effects of ECM stiffness were investigated using a hydrogel matrix as a model ECM. We made several hydrogel matrices of various stiffnesses (from 0.7–40 kPa) and observed that a hydrogel matrix with a stiffness of 4.47kPa is sufficient for TAZ activation and osteogenic differentiation. Furthermore, TAZ activation is induced by ERK and JNK activation, suggesting that ECM stiffness induces TAZ activation through ERK and JNK (Fig 7).

TAZ regulates MSC fate and is a mediator of Wnt signal [15,23] and was also identified as a mediator of mechanotransduction, which is the process by which cells sense and adapt to external forces and physical constraints. A stiff ECM stimulates the nuclear localization of TAZ/YAP through Rho activation and facilitates osteogenic differentiation [8]. McBeath et al. previously reported that Rho activation stimulates osteogenic differentiation [24]. In our study, we also observed that a ROCK inhibitor significantly decreased TAZ nuclear localization and osteogenic differentiation of MSCs on a 4.47 kPa hydrogel matrix (Fig 6). Next, we attempted to find other signaling components of the mechanotransduction pathway for TAZ activation. Interestingly, we observed that addition of an ERK or JNK inhibitor suppressed TAZ nuclear localization and osteogenic differentiation of MSCs on a stiff ECM (Figs 4 and 5), suggesting that the Rho signal facilitates TAZ activation through MAPK signaling. Interestingly, TAZ/YAP activation through MAPK was also previously observed by our group and others. We previously observed that FGF2-induced ERK activation increases the nuclear localization of TAZ [19]. In Drosophila, EGFR activates a TAZ paralogue (Yorkie) through the Ras-MAPK pathway [25]. YAP also functions as a critical transcriptional switch downstream of the oncogenic KRAS-MAPK pathway for neoplastic progression to pancreatic ductal adenocarcinoma [26]. These findings indicate that ERK activation plays an important role in TAZ/YAP activation, although the detailed activation mechanism is not yet known.

The importance of MAPK signaling in osteogenesis has been demonstrated in many other studies. MAPKs, including ERK and JNK, regulate osteogenic differentiation through transcription regulation [18]. Increased skeletal size and calvarial mineralization was observed in mice with constitutively active ERK [27]. ERK directly phosphorylates RUNX2, a master regulator of osteogenic differentiation, and activates ATF4 via RSK2, which stimulates osteogenic differentiation [28,29]. JNK inhibition blocks late-stage osteoblast differentiation, and overexpression of constitutively active MEKK2 and subsequent JNK1 activation is sufficient to enhance osteoblast activity [30]. In addition, JNK activates the transcription factors AP-1 and ATF2, which are involved in bone formation [31,32]. Thus, it seems that the MAPK-TAZ signaling axis in a stiff bone environment may provide an important signal for bone formation.

Taken together, our study demonstrates that a 4.47 kPa hydrogel activates TAZ and stimulates TAZ-mediated osteogenic differentiation through ERK and JNK. Thus, we suggest that ERK and JNK are important signaling mediators of stiff matrix-induced osteogenesis.

## Conclusions

We identified the minimal hydrogel matrix stiffness required for TAZ activation and osteogenic differentiation. The stiff hydrogel matrix stimulates ERK and JNK to activate TAZ and induce osteogenic differentiation. Thus, we suggest that ERK and JNK signaling is an important mediator of ECM stiffness-induced osteogenesis.

## Materials and Methods

### Cell culture

hMSCs were purchased from Lonza and cultured in DMEM supplemented with 10% fetal bovine serum (Hyclone). All cells were maintained at 37°C and 5% CO_2_.

### Preparation of hydrogel substrates

All procedures for the preparation of hydrogel substrates were described in a previous study [33]. In brief, 25-mm coverslips were coated with 0.1 M NaOH and APES (Sigma). The coverslips were washed with distilled H_2_O and then incubated in 0.5% glutaraldehyde for 30 min. Glutaraldehyde was removed, and the coverslips were air dried. Chloro-silanated glass slides were prepared by coating them with DCDMS (Sigma) and washing with distilled H_2_O. The gel mixture was prepared by mixing acrylamide and bis-acrylamide in the desired ratio. After the addition of a 1/100 total volume of APS and a 1/1000 total volume of TEMED to the gel mixture, 25 μL of gel solution was placed on a chloro-silanated glass slide and covered with an amino-silanated coverslip. After polymerization, the bottom glass slide was removed, and the gel-coverslip composite was placed in a 6-well plate and washed with distilled H_2_O.

### Antibodies

Anti-p-ERK (#9101), anti-ERK (#9102), anti-p-JNK (#9251), and anti-JNK (#9252) antibodies were purchased from Cell Signaling Technology. An anti-TAZ (#560235) antibody was purchased from BD Biosciences. An anti-vinculin (#ab18058) antibody was obtained from Abcam. An anti-GAPDH (#sc-32233) antibody was obtained from Santa Cruz Biotechnology.

### Immunocytochemistry

Human MSCs were seeded on hydrogel at a density of 1 × 10^4^/cm^2^. After 24 h, the cells were fixed with 2% formaldehyde for 15 min. After permeabilization with 0.3% Triton X-100, the cells were blocked with 5% normal goat serum for 1 h. The primary antibody was diluted in 1% BSA, and the cells were incubated with the diluted antibody at 4°C overnight. After three washes with PBS, a diluted secondary antibody was added to the specimens and incubated at room temperature for 2 h. The specimens were washed with PBS three times and then counterstained with Phalloidin to visualize F-actin and with DAPI to stain the nucleus. Images were acquired with a confocal microscope (CLSM 510META; Carl Zeiss).

### Osteogenic and adipogenic differentiation

For osteogenesis, human MSCs (hMSCs) were seeded on hydrogel at a density of 2 × 10^4^/cm^2^. After 24 h, the medium was changed to osteogenic differentiation medium (DMEM supplemented with 10% FBS, 50 μg/mL ascorbic acid, 0.1 μM dexamethasone, and 10 mM β-glycerophosphate). For adipogenesis, the hMSCs were incubated in adipogenic differentiation medium (DMEM supplemented with 10% FBS, 5 μg/mL insulin, 1 μM dexamethasone, and 1 μM troglitazone). In all cases, the differentiation media were replaced every two days.

### Alkaline phosphatase staining

Differentiated cells were fixed in 3.7% formaldehyde and incubated with the staining mixture (0.1 mg mL^-1^ naphthol AS-MX phosphate, 0.5% N,N-dimethylformamide, 2 mM MgCl_2_, and 0.6 mg mL^-1^ Fast Blue BB salt) for 30 min. After staining, images of the stained cells were acquired with a microscope and digital camera.

### Von Kossa staining

Differentiated osteoblasts were washed with PBS and fixed with 3.7% formaldehyde for 10 min. After wash with dH_2_O, cells were incubated in 2% silver nitrate under UV exposure for 30 min. Then cells were washed with dH_2_O, and incubated in 0.3% sodium thiosulfate for 5min. Cells were washed again with dH_2_O and air-dried.

### Oil-Red O staining

Differentiated adipocytes were washed with PBS and fixed with 3.7% formaldehyde for 30min. Then, cells were washed with PBS and incubated in 0.3% Oil-Red O in 60% isopropanol solution for 1 h. After wash with dH_2_O, cells were observed through microscopy.

### Immunoblot analysis

Cells were harvested by scraping into 1× SDS sample buffer. After a brief sonication step, the samples were heated at 95°C for 5 min, and then centrifuged to collect the cleared lysate (supernatant). Protein samples were separated by SDS-PAGE and transferred to a PVDF membrane. The blotted membrane was blocked with 5% skim milk in TBST for 1 h. Then, the primary antibody was diluted in TBST containing 5% BSA and was added to the membrane. After an overnight incubation at 4°C, the membrane was washed with TBST three times for 5 min each. The membrane was then incubated with the diluted secondary antibody for 1 h at room temperature. The target protein was detected by an ECL system (Ab signal; AbClon).

### Gene expression analysis by quantitative real-time PCR

Cells were harvested, and total RNA was prepared with TRIzol reagent (Invitrogen). cDNA was synthesized by M-MLV reverse transcriptase (Thermo). The cDNA samples were analyzed by quantitative real-time PCR (LightCycler480; Roche). The sequences of the primer sets used are listed in Table 1.

**Table 1:** Primers for qRT-PCR.

Gene	Direction	Sequence
*CTGF*	Forward	5′-CGACTGGAAGACACGTTTGG-3′
	Reverse	5′-CAGGTCTTGGAACAGGCG-3′
*CYR61*	Forward	5′-GAGTGGGTCTGTGACGAGGAT-3′
	Reverse	5′-GGTTGTATAGGATGCGAGGCT-3′
*DLX5*	Forward	5′-CTACAACCGCGTCCCAAG-3′
	Reverse	5′-GCCATTCACCATTCTCACCT-3′
*MSX2*	Forward	5′-CTACCCGTTCCATAGACCTGT-3′
	Reverse	5′-GAGAGGGAGAGGAAACCCTTT-3′
osteocalcin	Forward	5′-TGAGAGCCCTCACACTCCTC-3′
	Reverse	5′-ACCTTTGCTGGACTCTGCAC-3′
*RUNX2*	Forward	5′-AGAGGTACCAGATGGGACTGT-3′
	Reverse	5′-GGTAGCTACTTGGGGAGGATT-3′
adiponectin	Forward	5′-AAGGAGATCCAGGTCTTATTG-3′
	Reverse	5′-ACCTTCAGCCCCGGGTAC-3′
*aP2*	Forward	5′-GGCATGGCCAAACCTAACAT-3′
	Reverse	5′-TTCCATCCCATTTCTGCACAT-3′
*C/EBP* ***α***	Forward	5′-AAGGTGCTGGAGCTGACCAG-3′
	Reverse	5′-AATCTCCTAGTCCTGGCTCG-3′
*GAPDH*	Forward	5′- ACATCGCTCAGACACCATG-3′
	Reverse	5′-TGTAGTTGAGGTCAATGAAGGG-3′

### Luciferase reporter gene assay

hMSCs were transfected with the CTGF-luc (kindly provided by Guan Lab) or 6OSE2-luc plasmid by using Xtremegene HP transfection reagent. For the 6OSE2-luc gene assay, a myc-tagged Runx2 plasmid was co-transfected. In all experiments, a Renilla luciferase plasmid was co-transfected as an internal control. After 16 h of transfection, the cells were reseeded on the hydrogel, and inhibitors were added to the medium after cell attachment. Cells were maintained for 24 h and lysed with 1× passive lysis buffer (Promega). Lysates were analyzed with the Dual Luciferase Reporter Assay System (Promega) and a luminometer (GLOMAX, Promega). All samples were analyzed in triplicate.

### Statistical analysis

Data are shown as the mean ± SD. Experimental results were analyzed to confirm the significance of differences between groups by using Student’s t-test. (*p < 0.05, ***p < 0.005)

## Supporting Information

S1 Fig TAZ localization in cells on hydrogels with different degrees of stiffness. (A) hMSCs were plated on hydrogels with different degrees of stiffness (0.7, 4.47, 8.73, and 40 kPa). Cell adhesion and morphology were visualized by light microscopy. (B) TAZ localization in panel (A) was assessed by immunocytochemical analysis. DAPI was used to stain the nuclei. The results show nuclear localization of TAZ in cells 4.47, 8.73, and 40 kPa hydrogels. Thus, the transition of TAZ from the cytosol to the nucleus occurred in the range of 0.7–4.47 kPa.
(TIFF)

S2 Fig ECM stiffness regulated cell phenotype and cytoskeletal structure. (A) hMSCs were plated on 1.37 and 4.47 kPa hydrogels and stained with phalloidin and DAPI to detect F-actin and nuclei, respectively. On 1.37 kPa hydrogels, cells lost their normal spread phenotype and F-actin structure, which were well maintained in cells seeded on 4.47 kPa hydrogels. (B) Cell adhesion and morphology in panel (A) were visualized by light microscopy.
(TIFF)

S3 Fig TAZ knockdown blocks stiffness induced ostegenic marker genes expression. (A) hMSCs were infected with TAZ shRNA producing lentivirus and TAZ knockdown cells (Ti) were prepared. Lentivirus derived from a control vector was used for making control cells (Con). TAZ expression was analyzed by immunoblot analysis with the prepared cell lysates. (B) hMSCs in panel (A) were seeded on a 1.37 or 4.47 kPa hydrogel, and osteogenic differentiation was induced 24 h after seeding. At 6 days after differentiation, total RNAs were isolated and qRT-PCR analysis was assessed to see the expression of osteoblast marker genes *DLX5*, *MSX2*, Osteocalcin, and *RUNX2*. (*p < 0.05, **p < 0.01, ***p < 0.005, t-test).
(TIFF)

S4 Fig Inhibition of the ERK or JNK signal on 4.47 kPa hydrogel decreases TAZ expression. (A) hMSCs on a 4.47 kPa hydrogel were treated with 10 μM U0126 or 10 μM SP600125. After 12 h of treatment, total RNAs were prepared and qRT-PCR was assessed to analyze the transcription of *TAZ*. Gene expression was normalized to *GAPDH*. (B) Cell lysates in panel (A) were prepared and analyzed by immunoblotting. To assess the activity of ERK and JNK, phosphorylated ERK (p-ERK) and phosphorylated JNK (p-JNK) antibodies were used, respectively. As a control, total ERK and JNK protein was analyzed. The protein levels of TAZ was also analyzed with TAZ antibody. GAPDH was used as a loading control.
(TIFF)
