# Supplementary figures and images for: Extracellular Matrix Stiffness Regulates Osteogenic Differentiation through MAPK Activation
Source: PLoS One. 2015 Aug 11;10(8):e0135519. doi: 10.1371/journal.pone.0135519 (PMC4532446; doi:10.1371/journal.pone.0135519)

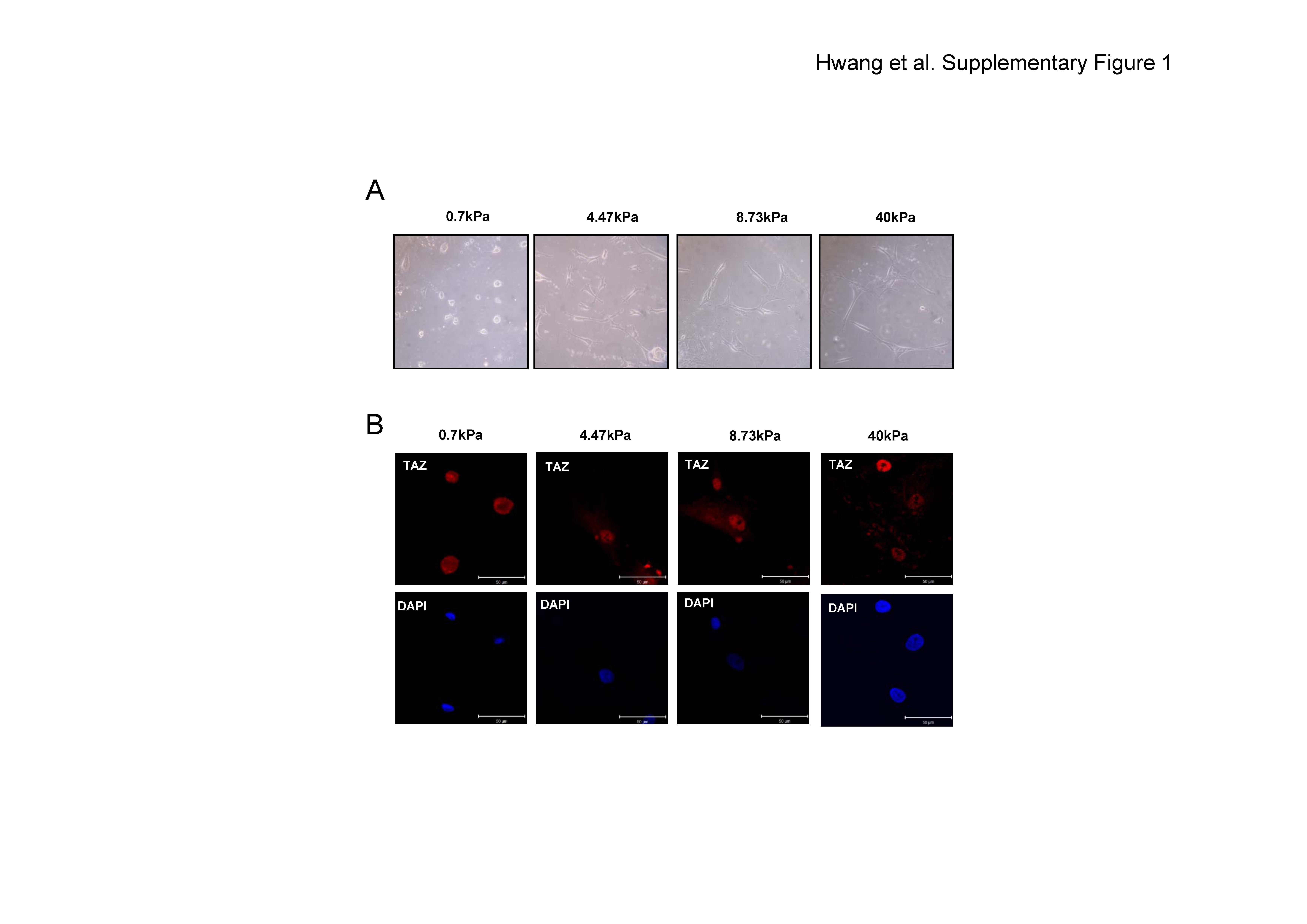

Supplement: S1 Fig — (A) hMSCs were plated on hydrogels with different degrees of stiffness (0.7, 4.47, 8.73, and 40 kPa). Cell adhesion and morphology were visualized by light microscopy. (B) TAZ localization in panel (A) was assessed by immunocytochemical analysis. DAPI was used to stain the nuclei. The results show nuclear localization of TAZ in cells 4.47, 8.73, and 40 kPa hydrogels. Thus, the transition of TAZ from the cytosol to the nucleus occurred in the range of 0.7–4.47 kPa. (TIFF) [file pone.0135519.s001.tiff]

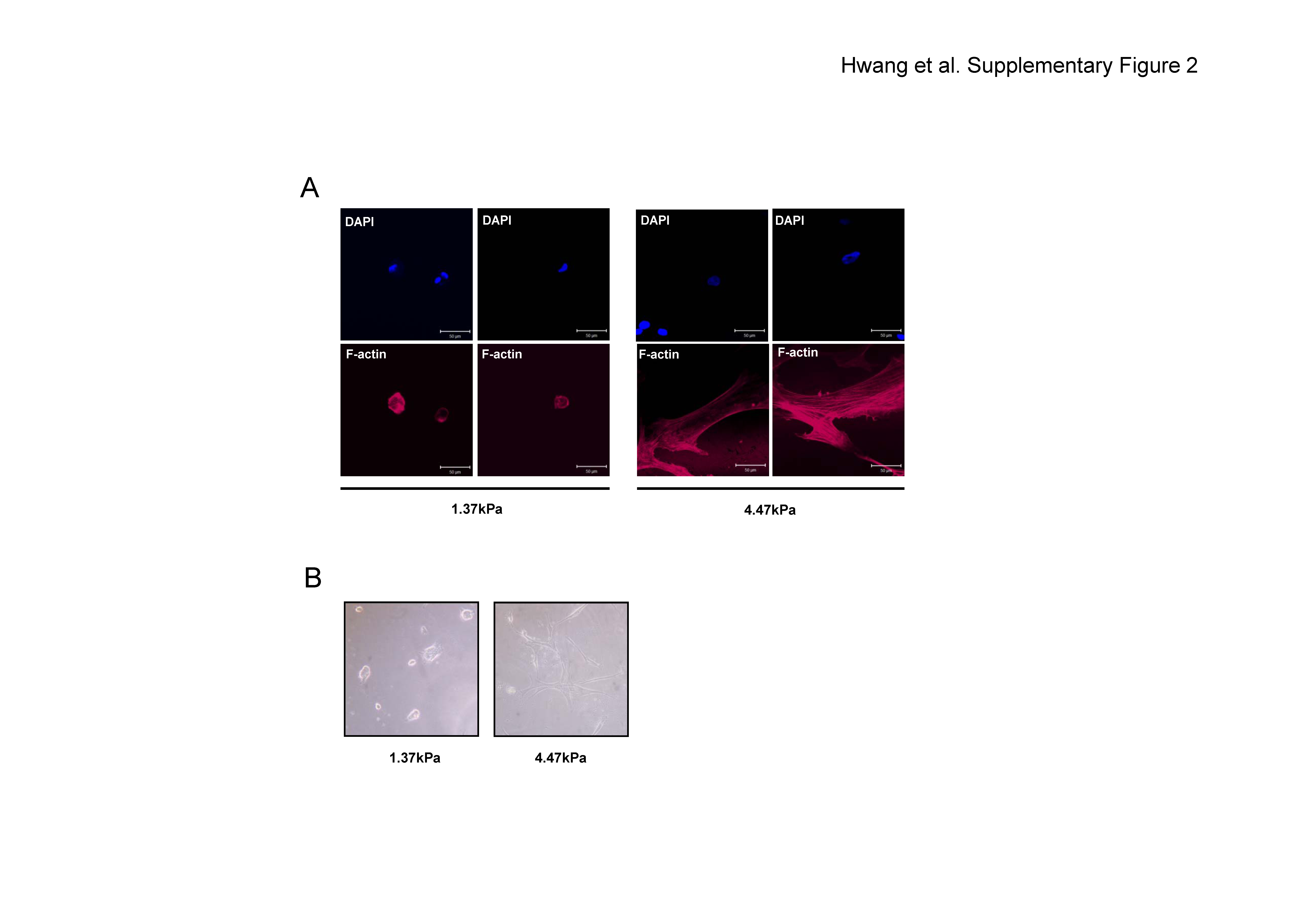

Supplement: S2 Fig — (A) hMSCs were plated on 1.37 and 4.47 kPa hydrogels and stained with phalloidin and DAPI to detect F-actin and nuclei, respectively. On 1.37 kPa hydrogels, cells lost their normal spread phenotype and F-actin structure, which were well maintained in cells seeded on 4.47 kPa hydrogels. (B) Cell adhesion and morphology in panel (A) were visualized by light microscopy. (TIFF) [file pone.0135519.s002.tiff]

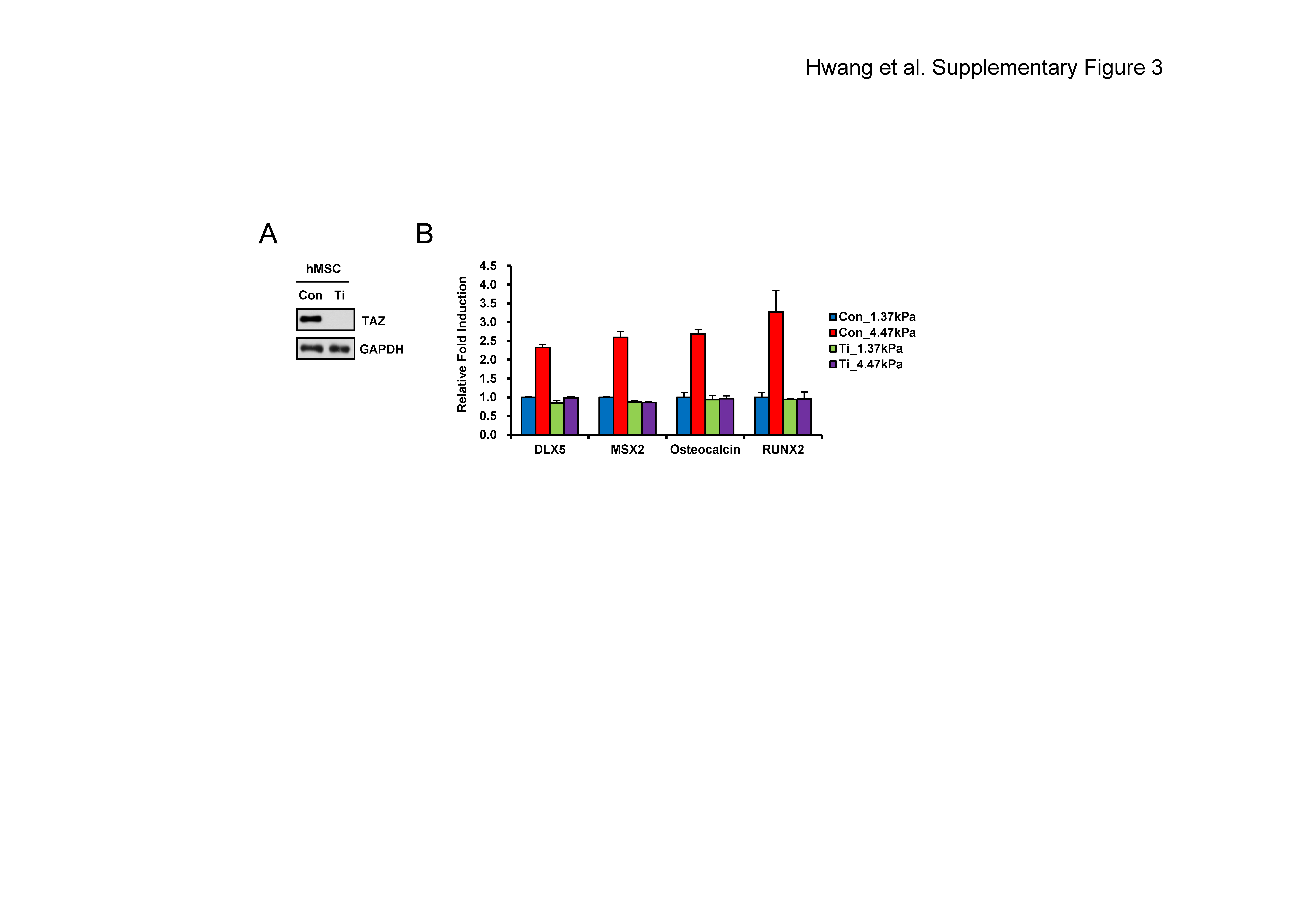

Supplement: S3 Fig — (A) hMSCs were infected with TAZ shRNA producing lentivirus and TAZ knockdown cells (Ti) were prepared. Lentivirus derived from a control vector was used for making control cells (Con). TAZ expression was analyzed by immunoblot analysis with the prepared cell lysates. (B) hMSCs in panel (A) were seeded on a 1.37 or 4.47 kPa hydrogel, and osteogenic differentiation was induced 24 h after seeding. At 6 days after differentiation, total RNAs were isolated and qRT-PCR analysis was assessed to see the expression of osteoblast marker genes DLX5, MSX2, Osteocalcin, and RUNX2. (*p < 0.05, **p < 0.01, ***p < 0.005, t-test). (TIFF) [file pone.0135519.s003.tiff]

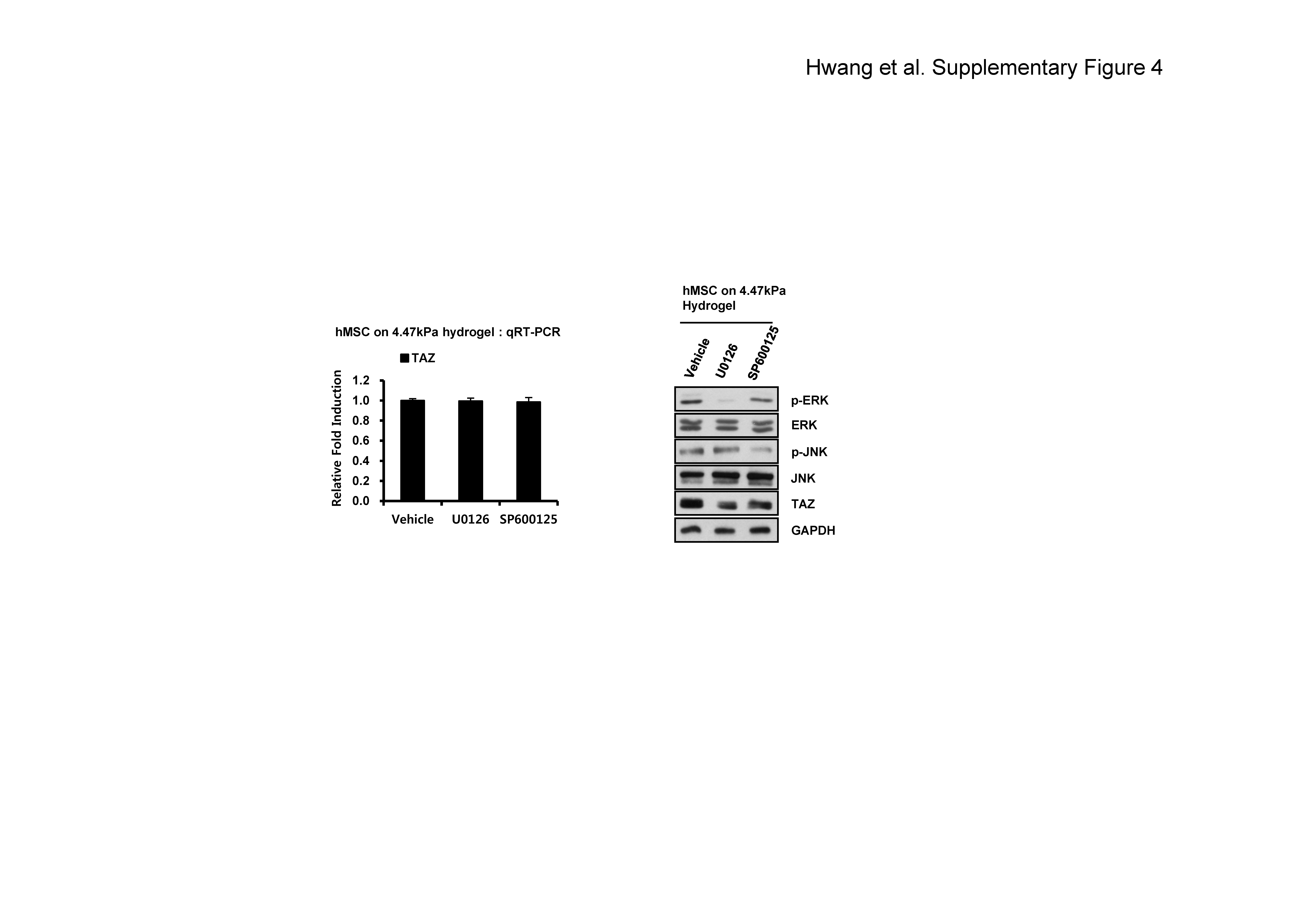

Supplement: S4 Fig — (A) hMSCs on a 4.47 kPa hydrogel were treated with 10 μM U0126 or 10 μM SP600125. After 12 h of treatment, total RNAs were prepared and qRT-PCR was assessed to analyze the transcription of TAZ. Gene expression was normalized to GAPDH. (B) Cell lysates in panel (A) were prepared and analyzed by immunoblotting. To assess the activity of ERK and JNK, phosphorylated ERK (p-ERK) and phosphorylated JNK (p-JNK) antibodies were used, respectively. As a control, total ERK and JNK protein was analyzed. The protein levels of TAZ was also analyzed with TAZ antibody. GAPDH was used as a loading control. (TIFF) [file pone.0135519.s004.tiff]
